# Serine deamination by human serine racemase synergizes with antibiotics to curtail the replication of *Chlamydia trachomatis*

**DOI:** 10.1016/j.jbc.2024.107350

**Published:** 2024-05-06

**Authors:** Patricia D. Mott, Arnold H. Zea, Jamiya Lewis, Oygul Mirzalieva, Ashok A. Aiyar

**Affiliations:** 1 Department of Biochemistry and Molecular Biology, Louisiana State University Health Sciences Center, New Orleans, Louisiana, USA; 2 Department of Microbiology, Immunology, & Parasitology, Louisiana State University Health Sciences Center, New Orleans, Louisiana, USA

**Keywords:** ammonia, serine, *Chlamydia*, *Chlamydia trachomatis*, bacterial metabolism, CRISPR/Cas9, serine deamination, serine racemase, serine dehydratase

## Abstract

The obligate intracellular bacterium, *Chlamydia trachomatis*, has evolved to depend on its human host for many metabolites, including most amino acids and three of the four nucleotides. Given this, it is not surprising that depletion of a single amino acid in the host cell growth medium blocks chlamydial replication. Paradoxically, supra-normal levels of some amino acids also block productive replication of *Chlamydia*. Here, we have determined how elevated serine levels, generated by exogenous supplementation, impede chlamydial inclusion development and reduce the generation of infectious progeny. Our findings reveal that human serine racemase, which is broadly expressed in multiple tissues, potentiates the anti-chlamydial effect of elevated serine concentrations. In addition to reversibly converting l-serine to d-serine, serine racemase also deaminates serine *via* β-elimination. We have determined that d-serine does not directly impact *Chlamydia*; rather, ammonia generated by serine deamination limits the productive chlamydial replication. Our findings imply that ammonia produced within host cells can traverse the chlamydial inclusion membrane. Further, this property of serine deaminase can be exploited to sensitize *Chlamydia* to concentrations of doxycycline that are otherwise not bactericidal. Because exogenously elevated levels of serine can be tolerated over extended periods, the broad expression pattern of serine racemase indicates it to be a host enzyme whose activity can be directed against multiple intracellular bacterial pathogens. From a therapeutic perspective, demonstrating host metabolism can be skewed to generate an anti-bacterial metabolite that synergizes with antibiotics, we believe our results provide a new approach to target intracellular pathogens.

Members of the order Chlamydiales are obligate intracellular bacteria that infect multiple host species. In humans, infections of conjunctival epithelia by *Chlamydia trachomatis* serovars A-C are etiologically associated with blindness, while genital epithelial infections by serovars D-K are sexually transmitted with implications on fertility and fetal health. Serovars L1-3 infect both epithelial cells and macrophages causing an invasive infection termed lymphogranuloma venereum (1). From an evolutionary perspective, their obligate intracellular life cycle has driven a reduction in the chlamydial genome, with the bacteria becoming dependent on their host cells for multiple metabolites including amino acids (2, 3) and three of the four nucleotides (4, 5).

Perturbations in host metabolic pathways that restrict the availability of these precursors within host cells affect chlamydial growth. For instance, we have shown that expression of the HPV-16 E6 and E7 oncogenes in the epithelial cell line, C-33A, reduces the availability of free intracellular tryptophan by impacting proteosome-dependent protein recycling (6). This decreased availability of tryptophan reduces bacterial replication and increases resistance to antibiotics such as doxycycline (*ibid*), whose mechanism depends on the availability of active protein translation as a target (7, 8). Previous studies have shown that the depletion or reduction of a single amino acid in the host-cell growth medium can restrict bacterial growth (2). Paradoxically, supplementation of host growth medium with supra-normal levels of specific amino acids also induces abnormalities in *C. trachomatis* development and infectious recovery. Supra-normal levels of phenylalanine, leucine, isoleucine, or methionine markedly reduce infectious recovery from HeLa cells (9); in this instance, elegant experiments by the group of Meyer revealed the block to result from competition for the chlamydial branched-chain amino acid transporter, BrnQ (10). Indeed, supplementing valine levels reverses the block imposed by high levels of the other branched-chain amino acids (10). These investigators also demonstrated that the addition of high levels of serine and threonine at the start of infection dramatically reduced the recovery of infectious progeny (9). However, this decrease was not reversed by the addition of valine leading to the suggestion that supra-normal levels of serine and threonine blocked chlamydial replication by competition for other metabolites or amino acids (10).

In this report, we evaluate an alternative hypothesis by which supra-normal levels of serine reduce the productive replication of *C. trachomatis*; specifically, that it is used as a substrate to generate a product deleterious to the productive replication of *C. trachomatis*. Our hypothesis was driven by our observation that chlamydial tryptophan synthase can catalyze a β-elimination reaction on serine to generate pyruvate and ammonia (11). Unlike other bacteria, or their host cells, *C. trachomatis* lacks commonly used enzymes for ammonia fixation, such as glutamine synthase, glutamate dehydrogenase, and glutamate synthase (https://www.genome.jp/dbget-bin/get_linkdb?-t+genes+gn:T02622), rendering this bacterium unusually sensitive to intracellular ammonia (11). The most prominent human enzymes that function as an l-serine ammonia lyase to produce ammonia are serine dehydratase (SDS) (https://www.proteinatlas.org/ENSG00000135094-SDS) (12) and serine dehydratase like 1 (SDSL) (12) (https://www.proteinatlas.org/ENSG00000139410-SDSL). Expression of SDS and SDSL is highly tissue-specific and restricted to hepatocytes and possibly some transformed cells (12, 13) (https://www.proteinatlas.org/ENSG00000139410-SDSL/tissue, https://www.proteinatlas.org/ENSG00000135094-SDS/tissue). Therefore, we searched for other human serine deaminating enzymes that are expressed more broadly, including mucosal tissues infected by *C. trachomatis*. We noticed that the supra-normal levels of serine and threonine that inhibited chlamydial growth coincided with the level of these amino acids used to supplement media in studies investigating the ability of human serine racemase (SRR) to degrade these amino acids *via* β-elimination (14, 15, 16). The products of β-elimination by SRR are pyruvate and ammonia (for serine), and α-ketobutyrate and ammonia (for threonine). Like bacterial tryptophan synthase, SRR is a PLP enzyme; the identified β-elimination mechanism for SRR on serine closely parallels the mechanism proposed for bacterial tryptophan synthases (17). Within cells and *in vitro*, SRR catalyzes two reactions: (1) the racemization of l-serine to d-serine (14, 18), with the latter functioning as a co-agonist of the neuronal NMDA receptor (19); and (2) a β-elimination reaction on both enantiomers of serine to generate ammonia and pyruvate (14, 15). Elegant mechanistic studies conducted *in vitro* on SRR reveal the latter reaction to predominate over the former (16, 17, 20), giving us cause to wonder whether the capacity of SRR to generate ammonia during high availability of serine, and/or threonine, was a mechanism by which high-levels of these amino acids interfered with chlamydial replication. In addition to the ammonia product of SRR, d-serine may also negatively impact chlamydial replication because d-serine modulates gene expression in some bacterial genera (21, 22, 23, 24, 25) and interferes with peptidoglycan synthesis in others (26, 27).

The validity of our hypothesis hinges on the expression of SRR in tissues relevant to chlamydial infection. Therefore, we mined the Human Protein Atlas to evaluate pre-existing datasets on the tissue expression pattern of SRR (12) (https://www.proteinatlas.org/ENSG00000167720-SRR) at the RNA and protein level. Although its d-serine product functions as a neurotransmitter, SRR expression shows little tissue specificity at the RNA or protein level (12, 28, 29) (https://www.proteinatlas.org/ENSG00000167720-SRR/tissue). Pertinent to our hypothesis, the levels of SRR expression seen at multiple tissues including mucosal epithelia are similar to, or even exceed, levels observed in the brain. Immunohistochemistry conducted using the same antibody used to detect SRR expression in neural sections reveals it to be particularly elevated in the columnar epithelial cells in cervical sections (12) (https://www.proteinatlas.org/ENSG00000167720-SRR/tissue/cervix#img), which are believed to be the primary cells infected by *C. trachomatis* in the female reproductive tract (30).

We confirmed the expression of SRR in multiple human cell-lines of cervical origin. Of these, we conducted further experiments with the HeLa cell-line, because supra-normal levels of serine were first shown to limit productive chlamydial replication in this cell-line (9). CRISPR/Cas9 was used to stably ablate the SRR gene in HeLa, creating a new cell-line termed HeLa ΔSRR. Experiments were conducted comparing the replication of *C. trachomatis* in HeLa and HeLa ΔSRR. Outcomes from these studies strongly support a role for SRR in restricting the productive replication of *C. trachomatis*, *via* serine β-elimination. Further, the growth restrictive effect of serine supplementation synergized strongly with the effect of doxycycline on *C. trachomatis*. Our findings reveal a novel interaction between host amino acid metabolism with antibiotic function that can be exploited against infections by *Chlamydia* and other intracellular pathogens.

## Results

### Multiple human cervical epithelial cell lines express SRR

**Figure 1:**
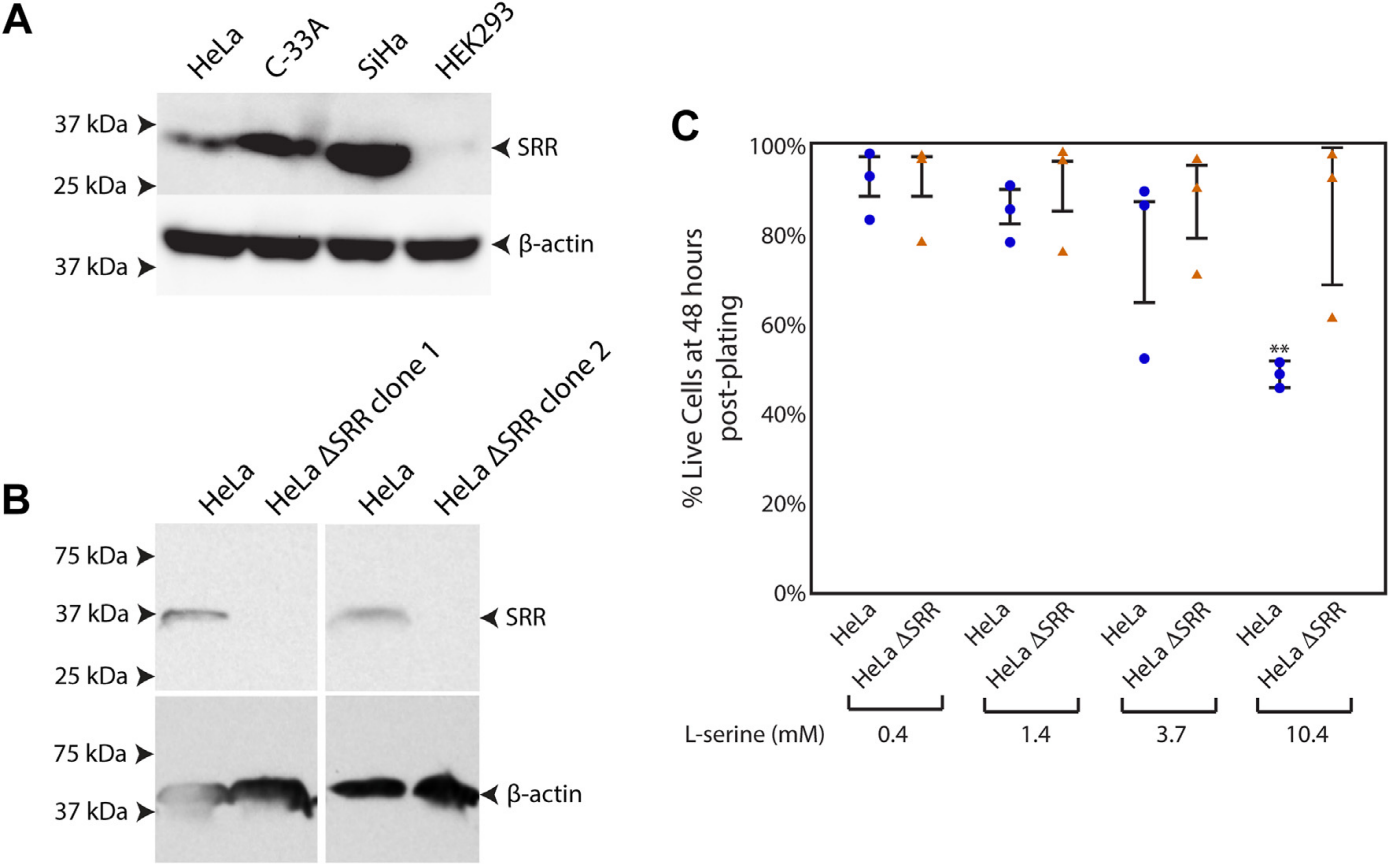
**Expression of serine racemase (SRR) in cervical origin cell lines, and generation of HeLa ΔSRR cells.***A*, the expression of SRR was evaluated in the cervical origin cell lines HeLa, C-33A, and SiHa, along with the adrenal/neuronal origin cell line HEK-293. Cell extracts were separated by SDS-PAGE, followed by immunoblot analysis using a monoclonal antibody specific for SRR. A parallel immunoblot was analyzed for the expression of β-actin as a control. The migration of molecular weight markers of the indicated size is highlighted by *arrowheads* on the *left* of each blot. Both SRR and β-actin migrated at their anticipated sizes. *B*, CRISPR/Cas9 was used to knock out the SRR gene in HeLa cells. Clones were selected using puromycin and screened for SRR expression by immunoblot as described in (*A*). Blots were stripped and re-probed to assess β-actin expression as a control. The two HeLa ΔSRR clones indicated were pooled and expanded and used for the experiments in this report. *C*, HeLa and HeLa ΔSRR cells were exposed to media supplemented with l-serine at the indicated concentrations for 48 h at which time cell survival was assessed using MTS assays. Unlike HeLa ΔSRR cells, HeLa cells could not tolerate l-serine supplementation at 10.4 mM. Two asterisks indicate a *p*-value < 0.01, using the Wilcoxon rank sum test, while comparing HeLa and HeLa ΔSRR cells exposed to 10.4 mM l-serine.

When added to cell culture media at supra-normal levels of 5 to 10 mM, serine and threonine dramatically reduce productive infection of HeLa cells by *C. trachomatis* serovar L2 (9). We have previously demonstrated that chlamydial tryptophan synthase can generate ammonia *via* a β-elimination reaction on serine, and when it does so, severely restricts the production of infectious bacteria (11). Therefore, we wondered whether high levels of exogenously added serine or threonine restricted the replication of *chlamydia* in HeLa cells by serving as a substrate for a host enzyme that functioned as an l-serine ammonia lyase (EC 4.3.1.17) and/or l-threonine ammonia-lyase (EC 4.3.1.19). We considered human serine dehydratase (gene symbol SDS) as a likely candidate as it has both these activities. However, serine dehydratase is almost exclusively expressed in hepatocytes, and pertinently, we were unable to detect its expression in HeLa and other epithelial cell lines of cervical origin by PCR or immunoblot (data not shown). Having eliminated serine dehydratase as a candidate, we considered serine racemase (gene symbol SRR) as an alternative for several reasons. First, SRR is active as a serine and threonine ammonia-lyase when ectopically expressed in HEK-293 cells (15, 19). The ability of SRR to generate pyruvate and ammonia from serine is also clearly observed in enzymatic studies performed using purified SRR *in vitro* (16, 17). Indeed, the latter studies reveal SRR’s capacity to produce ammonia *via* β-elimination to be several-fold more active than its ability to racemize serine (summarized in (17)). Second, unlike serine dehydratase, datasets in the Human Protein Atlas reveal SRR to be expressed broadly in multiple tissues, including mucosal surfaces associated with chlamydial infections such as the eye and the female reproductive tract. Therefore, we tested whether we could detect the expression of SRR in several human cell lines of cervical origin, including HeLa (ATCC CCL-2), C-33A (ATCC HTB-31), and SiHa (ATCC HTB-35). While all these lines are of cervical origin, they differ in that HeLa and SiHa are positive for HPV-18 and HPV-16 respectively, while C-33A is HPV-negative. HEK-293 (ATCC CRL-1573), which arose by adenovirus transformation of neuronal/adrenal lineage cells (31), was also included in this analysis. Cell extracts were evaluated by immunoblot using a rabbit monoclonal antibody specific for SRR. Parallel blots were probed with a monoclonal antibody against β-actin as a loading control. The results, shown in Figure 1*A*, indicate SRR to be expressed in all the cervical origin cell lines but not in HEK-293 cells. This result fits previous observations in which SRR activity was not detected, or present at very low levels, in HEK-293 cells (15, 19). Because SRR met our criteria of being a host enzyme with l-serine ammonia-lyase activity that is expressed in HeLa cells, we decided to evaluate whether it was necessary to potentiate the effect of high levels of serine against *C. trachomatis*.

Using plasmids described in the Experimental procedures, CRISPR/Cas9 was used to create a stable derivative of HeLa in which SRR was knocked out (HeLa ΔSRR). When evaluated by immunoblot, we were unable to detect the expression of SRR in HeLa ΔSRR cells (Fig. 1*B*). MTS assays indicated no difference in the proliferation or survival rate of HeLa ΔSRR relative to HeLa cells when grown in DMEM, which contains 0.4 mM l-serine (Fig. 1*C*). When grown for 48 h in DMEM containing 1.4 mM and 3.7 mM l-serine, the survival of HeLa cells was slightly impaired relative to HeLa ΔSRR, although this decrease was not significant. However, the survival of HeLa cells was significantly impaired when grown for 48 h in DMEM containing 10.4 mM l-serine (Fig. 1*C*); therefore, this concentration was not used in the experiments described below. No such effect was observed for HeLa ΔSRR cells (*ibid*).

Previous studies by Inoue *et al.* (32), performed using epithelial cells from wild-type mice or SRR knockout mice revealed that free intracellular l-serine levels were decreased in knockout epithelial cells relative to wild-type cells. Therefore, we used their procedure to measure intracellular l-serine levels in HeLa and HeLa ΔSRR cells. These data are summarized in Table 1. As reported by these authors, we were unable to detect free l-serine for cells grown in normal DMEM, containing 0.4 mM l-serine. Free l-serine was detected when cells were grown in DMEM containing 1.4 mM or 3.7 mM l-serine, and revealed that under both conditions the levels of l-serine were lower in HeLa ΔSRR cells. While no reason has been proposed for this decrease (32), it is likely that SRR plays a role in maintaining free l-serine homeostasis.

**Table 1:** Free l-serine levels are higher in HeLa relative to HeLa ΔSRR cells

L-serine in DMEM (mM)	HeLa (μM)^a^	HeLa ΔSRR (μM)^a^
0.4	N.D.^b^	N.D.^b^
1.4	107 ± 53 μΜ	62 ± 21 μΜ (*p* < 0.05)^c^
3.7	441 ± 47 μΜ	183 ± 78 μM (*p* < 0.01)^c^

a L-serine values were measured as described in the Experimental Procedures using the HPLC procedure of Inoue *et al.* (32).

b Free l-serine could not be detected when cells were grown in DMEM not supplemented with serine.

c *p*-values were obtained using the Wilcoxon Signed Rank Test as described in the experimental procedures.

### Higher levels of D-serine and ammonia are detected in supernatants from HeLa relative to HeLa ΔSRR

**Figure 2:**
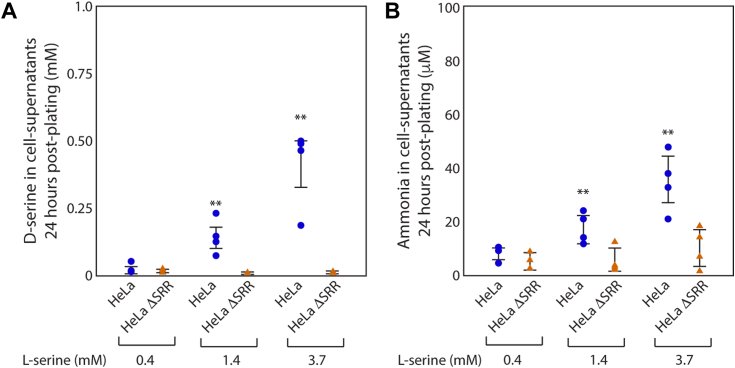
**HeLa ΔSRR cells do not produce d-serine or ammonia in response to l-serine supplementation in media.***A*, HeLa and HeLa ΔSRR cells were grown in DMEM containing l-serine at the indicated concentrations for 24 h prior to assessing the level of d-serine in cell supernatants as described in the Experimental procedures. D-serine was observed in HeLa cell supernatants during serine supplementation, but not in supernatants from HeLa ΔSRR cells. *B*, the level of ammonia/ammonium in cell supernatants from HeLa or HeLa ΔSRR cells was assessed after 24 h of growth in DMEM containing the indicated levels of l-serine. HeLa cell supernatants contained significantly higher levels of ammonia/ammonium when DMEM was supplemented with l-serine. Two *asterisks* indicate a *p*-value < 0.01, using the Wilcoxon rank sum test, while comparing HeLa and HeLa ΔSRR cells exposed to DMEM containing the same concentration of l-serine.

The level of d-serine in supernatants from HeLa and HeLa ΔSRR cells, when grown in DMEM with varying amounts of l-serine, was evaluated to determine whether the absence of SRR in the latter coincided with a lack of d-serine in the media. D-serine was measured as described in the Experimental procedures using a kit that employs a d-serine dehydratase from *Saccharomyces cerevisiae*. Preliminary experiments indicated the presence of d-serine levels in cell supernatants from both cell lines. Control experiments revealed the source of d-serine to be calf serum (data not shown), consistent with previous reports indicating the presence of various d-isoform amino acids in serum (33, 34). Therefore, DMEM supplemented with triple-dialyzed calf serum was used for the experiments in this report. As shown in Figure 2*A*, levels of d-serine were significantly higher in HeLa cells relative to HeLa ΔSRR when both cell lines were grown in DMEM containing elevated levels of l-serine. When grown in DMEM containing 1.4 mM l-serine, 48-h supernatants from HeLa cells had an average of 0.17 mM d-serine, an increase of approximately 6-fold over d-serine levels observed when HeLa cells were grown in control DMEM (Fig. 2*A*). Supernatants of HeLa cells grown in DMEM containing 3.7 mM l-serine had approximately 20-fold higher levels of d-serine relative to HeLa cells grown in control media (*ibid*). Under both these conditions, d-serine levels in HeLa ΔSRR supernatants did not change from levels observed during control growth DMEM.

Having confirmed that d-serine levels were diminished to the background in HeLa ΔSRR cells, we queried whether this was also true of ammonia levels. Ammonia was measured as described in the Experimental procedures using a kit that relies on the ability of glutamate dehydrogenase to generate glutamate using α-ketoglutarate and ammonium as substrates. As shown in Figure 2*B*, when grown in DMEM containing 1.4 mM or 3.7 mM l-serine, 48-h supernatants from HeLa cells contained significantly higher levels of ammonia relative to HeLa ΔSRR supernatants. When DMEM contained 1.4 mM l-serine, supernatants had an average of 18.7 μM ammonia *versus* 6.2 μM for HeLa ΔSRR. Similarly, ammonia levels were 39 μM and 9.7 μM when HeLa and HeLa ΔSRR were grown in DMEM containing 3.7 mM l-serine.

### Growth media with elevated l-serine levels impacts the productive replication of *C. trachomatis* serovar L2 in HeLa but not HeLa ΔSRR

**Figure 3:**
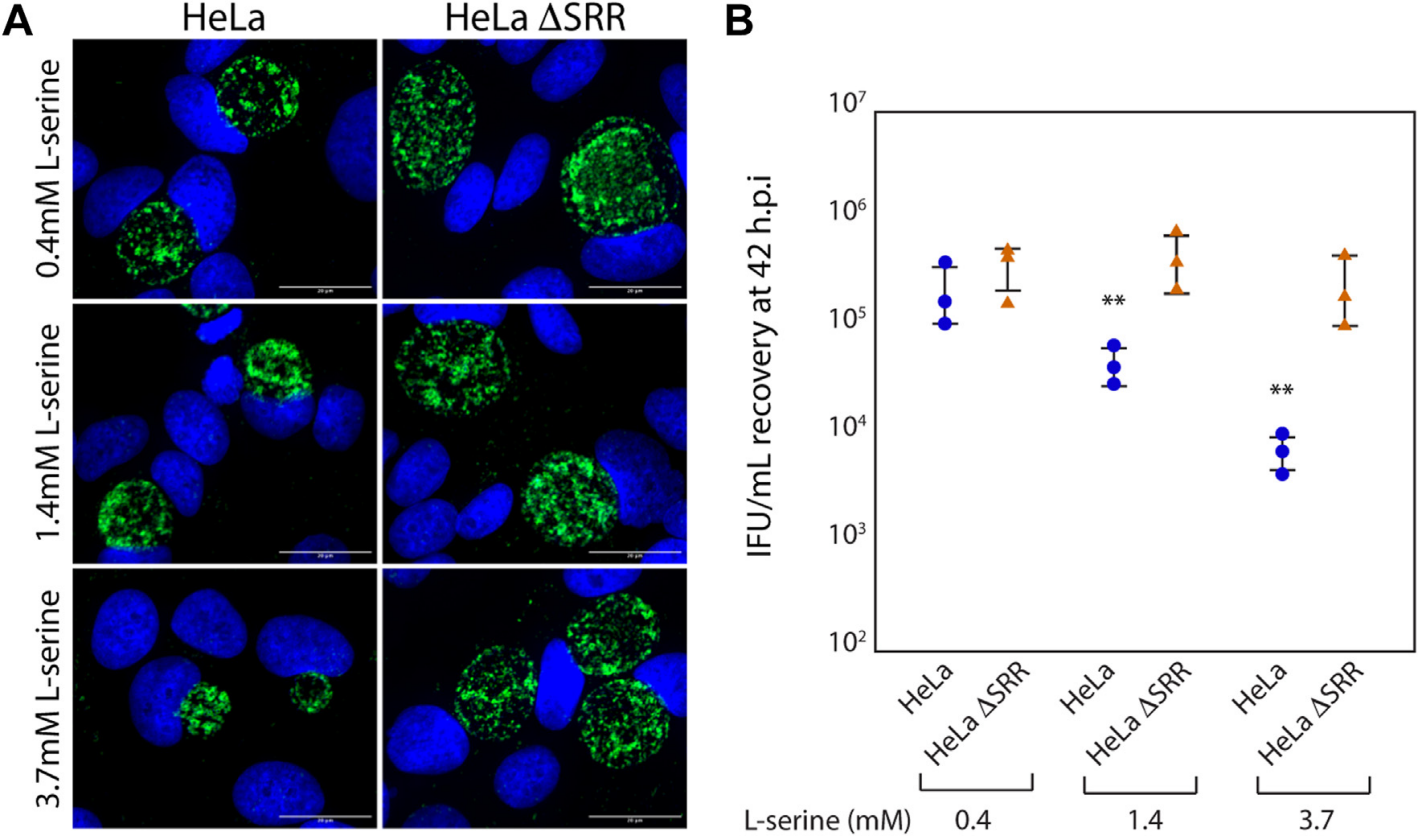
**L-serine supplementation restricts the growth of *C. trachomatis* serovar L2 (CT/L2) in HeLa but not HeLa ΔSRR cells.***A*, HeLa or HeLa ΔSRR cells were infected with CT/L2 at an m.o.i of 0.3. Infected cells were exposed to DMEM containing the indicated concentration of l-serine for 42 h after which inclusion morphology was assessed by indirect immunofluorescence as indicated in the methods. Inclusions are pseudo-colored *green*, while host cell nuclei, stained with DAPI, are pseudo-colored blue. The scale bar at the bottom right corner of each image indicates a distance of 20 μm. *B*, primary infections of HeLa and HeLa ΔSRR cells, exposed to DMEM containing the indicated concentrations of l-serine, were assessed for recovery of infectious units (IFUs) at 42 h.p.i as described in the Experimental procedures. Two *asterisks* indicate a *p*-value < 0.01, using the Wilcoxon rank sum test, while comparing CT/L2 infections of HeLa and HeLa ΔSRR cells exposed to DMEM containing the same concentration of l-serine.

HeLa and HeLa ΔSRR cells were infected with *C. trachomatis* L2/434/Bu, referred to as CT/L2, at a multiplicity of infection (m.o.i) of 0.3 as described in the Experimental procedures. Cells were fixed and stained at 42 h post-infection (h.p.i) (Fig. 3*A*) or extracted in SPG to evaluate the recovery of infectious units (IFUs) (Fig. 3*B*). CT/L2 formed smaller inclusions in HeLa relative to HeLa ΔSRR under all three conditions tested (Fig. 3*A* and Table S1). This effect was particularly pronounced when infected cells were grown in DMEM containing 3.7 mM l-serine. At this concentration of l-serine, the average inclusion area in HeLa cells was 33.12 μm^2^, relative to an average area of 227.07 μm^2^ observed for HeLa ΔSRR cells. It should also be noted that while inclusion sizes did decrease in HeLa ΔSRR cells at higher concentrations of l-serine, this decrease was far more pronounced in HeLa cells (Table S1). The l-serine concentration-dependent reduction in inclusion size observed in HeLa cells was paralleled by a significant decrease in IFU recovery at 42 h.p.i (Fig. 3*B*). IFU recovery from HeLa cells was reduced by approximately 0.5 logs at 1.4 mM l-serine and 1.5 logs at 3.7 mM l-serine. In contrast to these observations, 1.4 mM l-serine did not significantly decrease inclusion size in HeLa ΔSRR cells (Fig. 3*A*) or impact IFU recovery at 42 h.p.i (Fig. 3*B*). A significant, but minor, decrease in average inclusion size was observed when infected HeLa ΔSRR cells were grown in DMEM containing 3.7 mM l-serine (Fig. 3*A*, Table S1), with no significant decrease in IFU recovery at 42 h.p.i (Fig. 3*B*).

### Supplementing HeLa ΔSRR cell growth media with D-serine or pyruvate does not affect the productive replication of *C. trachomatis* serovar L2

**Figure 4:**
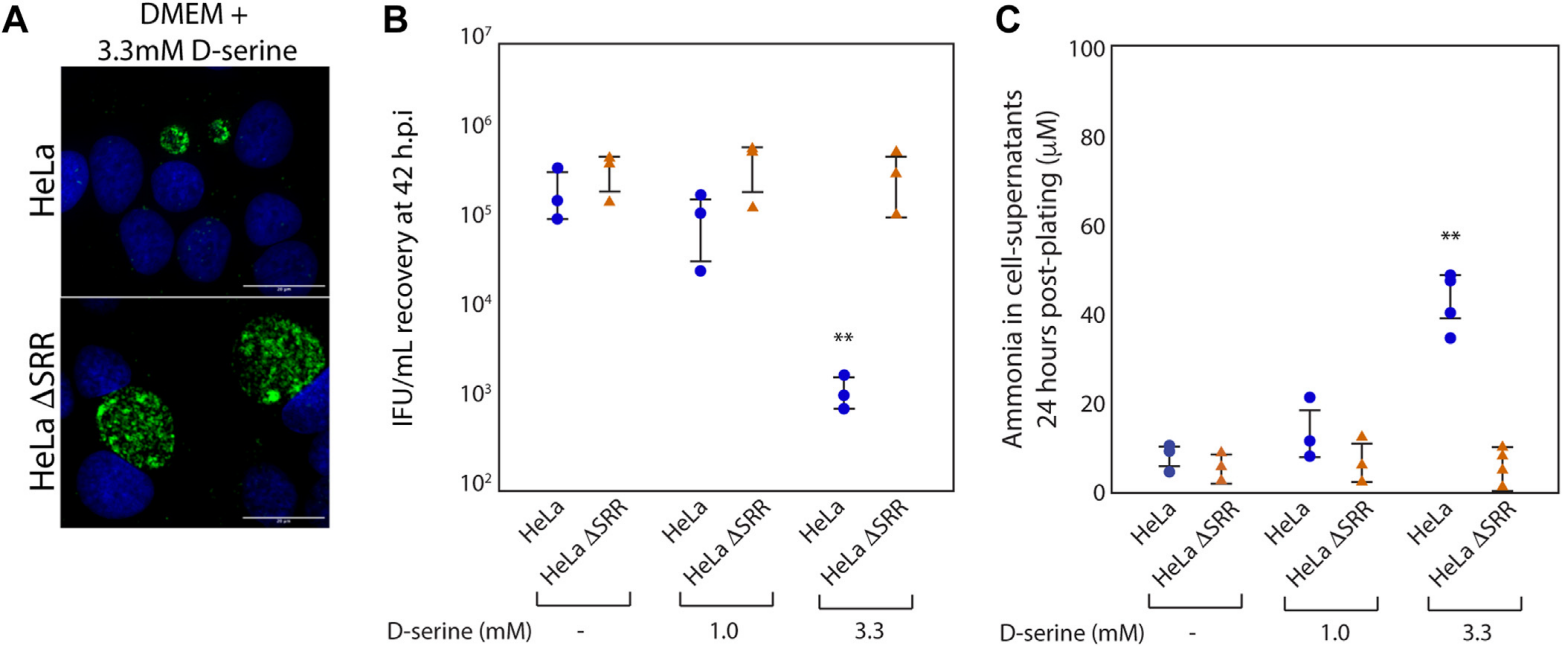
**SRR’s ability to racemize l-serine to d-serine is not the mechanism by which it restricts replication of *C. trachomatis*.***A*, HeLa and HeLa ΔSRR cells were infected with CT/L2 at an m.o.i of 0.3. Infected cells were exposed to DMEM containing 3.3 mM d-serine for 42 h at which point primary inclusions were visualized as described in Figure 3. At this concentration, d-serine did not affect inclusion morphology in HeLa ΔSRR cells, while inclusions in HeLa cells were much smaller. The scale bar at the bottom right corner of each image indicates a distance of 20 μm. *B*, primary infections of HeLa and HeLa ΔSRR cells, exposed to DMEM containing the indicated concentrations of d-serine, were assessed for recovery of infectious units (IFUs) at 42 h.p.i as described in the Experimental procedures. Corroborating the smaller inclusion size observed in (*A*), IFU recovery from HeLa cells exposed to 3.3 mM d-serine was significantly lower than IFU recovery from HeLa ΔSRR cells. *C*, supernatant ammonia levels were assessed for HeLa and HeLa ΔSRR cells at 24 h post-exposure to the indicated level of d-serine in DMEM. HeLa cells exposed to DMEM containing 3.3 mM d-serine displayed significantly higher levels of ammonia/ammonium in cell supernatants relative to HeLa ΔSRR cell supernatants. Two asterisks indicate a *p*-value < 0.01, using the Wilcoxon rank sum test, HeLa and HeLa ΔSRR cells exposed to DMEM containing the same concentration of d-serine.

Informed by our previously published studies (11), the experiments described above are interpreted to indicate that ammonia generated during serine deamination by serine racemase restricts the growth of *C. trachomatis*. However, pyruvate is also produced during serine deamination; additionally, serine racemase also catalyzes the racemic conversion of l-serine to d-serine (17), leading us to test their effects on the growth of *C. trachomatis*. D-serine is known to alter gene expression in some bacteria (22, 23, 24), and there have been reports of d-serine reducing bacterial survival by impacting peptidoglycan synthesis (26, 27). Because HeLa ΔSRR does not produce d-serine to a detectable level, we directly tested whether the addition of d-serine would impact the replication of CT/L2 in HeLa ΔSRR cells. Concurrently, we also evaluated the effect of d-serine supplementation on CT/L2 replication in HeLa cells. As shown in Figure 4*A*, supplementing media with 1 mM d-serine did not impact CT/L2 IFU recovery in either HeLa ΔSRR or HeLa cells. A higher level of d-serine, 3.3 mM, significantly reduced CT/L2 IFU recovery in HeLa cells, but not HeLa ΔSRR (Fig. 4*A*), with a concomitant reduction in inclusion size (Fig. 4*B*). Because l-serine and d-serine can be used as substrates for serine deamination by serine racemase (14, 16, 20), we measured ammonia in supernatants from HeLa and HeLa ΔSRR cells (Fig. 4*C*). Consistent with d-serine reducing CT/L2 IFU recovery in HeLa, but not HeLa ΔSRR, only supernatants from the former contained elevated levels of ammonia when media was supplemented with 3.3 mM d-serine. We interpret these data to indicate that the d-serine product of serine racemase activity does not impact CT/L2 replication. Further, these data reiterate the role of the ammonia product of serine deamination in restricting the intracellular growth of *C. trachomatis*.

We also tested whether pyruvate, the other product of serine β-elimination, affected *C. trachomatis* inclusion development and IFU recovery. DMEM normally contains 1 mM pyruvate; it has previously been shown that extended exposure of human cell lines, including HeLa, to 5 mM pyruvate decreases their rate of division as assayed throughout 4 to 5 days, while simultaneously repressing the expression of genes necessary for cell proliferation (35). These same studies revealed a minimal effect on cell proliferation when the exposure to high levels of pyruvate was for 48 h and less (35), *that is*, the time frame of experiments evaluating the replication of *C. trachomatis*. Therefore, we tested whether the addition of pyruvate would impact inclusion development and IFU recovery from CT/L2 infected HeLa and HeLa ΔSRR cells. Supplementing DMEM with an additional 1 mM pyruvate did not affect CT/L2 inclusion development or IFU recovery from either cell line (data not shown). However, when infected HeLa cells were grown media supplemented with an additional 3.3 mM pyruvate, a modest reduction in CT/L2 inclusion size (Fig. S1*A*) and IFU recovery (Fig. S1*B*) was observed. Both these effects were not observed in HeLa ΔSRR cells. We speculate that pyruvate is a substrate for serine biosynthesis in both cell types (36), but that the resulting serine is deaminated only in HeLa cells.

### Sub-inhibitory levels of serine synergize with sub-inhibitory concentrations of doxycycline to reduce CT/L2 replication in HeLa but not HeLa ΔSRR cells

Bacteria have multiple mechanisms to assimilate ammonia that is generated within them or that arises from an external source. The predominant enzymes used for direct ammonia assimilation include glutamine synthetase, glutamate dehydrogenase, and glutamate synthase, with NAD synthetase, carbamoyl phosphate synthetase, and asparagine synthetase used less frequently (37). *C. trachomatis* lacks all these enzymes, possibly increasing its sensitivity to raised ammonia levels relative to other bacteria. While the identity of chlamydial enzymes that can assimilate ammonia is unknown, we hypothesized that assimilating higher levels of ammonia will require more molecules of these enzymes. Therefore, we tested whether restricting protein synthesis using doxycycline would increase chlamydial sensitivity to raised serine levels in media.

**Figure 5:**
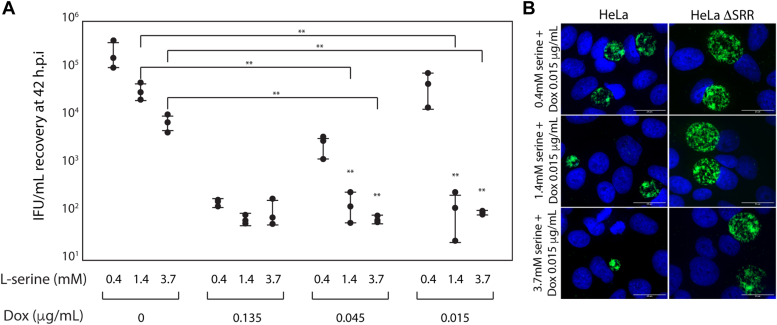
**L-serine supplementation synergizes with sub-inhibitory concentrations of doxycycline.***A*, HeLa cells were infected with CT/L2 at an m.o.i of 0.3. Post-infection, cells were exposed to DMEM containing the indicated concentration of l-serine alone, or together with the indicated concentration of doxycycline. IFU recovery was assessed at 42 h.p.i as indicated in the methods. The data for CT/L2 IFU recovery in the absence of doxycycline is reproduced from Figure 3 to facilitate visual comparisons indicating synergy between l-serine supplementation and sub-inhibitory concentrations of doxycycline. Two sets of statistical comparisons are indicated. First, for any single doxycycline concentration, two asterisks directly above a column indicate a comparison between the l-serine concentration for that sample *versus* an l-serine concentration of 0.4 mM. Second, brackets above the bar indicate a comparison between samples exposed to the same l-serine concentration but different doxycycline concentrations. Two *asterisks* indicate a *p*-value of < 0.01 as measured using the Wilcoxon rank sum test for the indicated comparison. *B*, primary inclusions formed when HeLa or HeLa ΔSRR cells were infected with CT/L2 at an m.o.i of 0.3, and exposed to the indicated concentration of l-serine along with doxycycline at 0.015 μg/ml. The scale bar at the bottom right corner of each image indicates a distance of 20 μm.

As reported previously, the minimum bactericidal concentration (MBC) of doxycycline (Dox) against CT/L2 in HeLa cells was observed to be 1 to 2 μg/ml (data not shown). In normal DMEM, a Dox concentration of 0.135 μg/ml reduced IFU recovery by approximately 3 logs (Fig. 5*A*). In normal DMEM, a Dox concentration of 0.015 μg/ml had no significant effect on CT/L2 IFU recovery (*ibid*). As shown in Figure 3, increasing media l-serine levels to 1.4 mM reduced CT/L2 IFU recovery by approximately 0.5 log. However, raising l-serine levels to 1.4 mM while concomitantly exposing infected HeLa cells to Dox 0.015 μg/ml reduced IFU recovery by ∼3-logs (Fig. 5*A*). Consistent with the decrease in IFU recovery, CT/L2 formed smaller inclusions in the presence of Dox 0.015 μg/ml when media levels of l-serine were raised (Fig. 5*B*). In contrast to observations made in HeLa cells, higher levels of l-serine did not increase the doxycycline sensitivity of CT/L2 infections in HeLa ΔSRR cells (data not shown, and Fig. 5*B*). In summary, these data indicate that when exposed to higher intracellular levels of ammonium, the sensitivity of CT/L2 to doxycycline is increased by approximately 1 log.

## Discussion

As an obligate intracellular bacterium, *Chlamydia* has evolved to lack multiple biosynthetic pathways, including those for most amino acids (2, 3), and three of the four nucleotides (4, 5). A search of the Kyoto Encyclopedia of Genes and Genomes (KEGG) (38, 39, 40) indicates that *C. trachomatis* also lacks enzymes commonly employed to assimilate the toxic metabolite ammonia (https://www.genome.jp/dbget-bin/get_linkdb?-t+genes+gn:T02622) (40). Previously, using a genital serovar of *C. trachomatis*, we have shown that forcing the expression of a functional tryptophan synthase in an indole-free environment results in serine deamination, with the ammonia by-product of deamination being bactericidal to *Chlamydia* (11). There are phenotypic parallels between our findings and those published by Al-Younes *et al.* (9), who demonstrated that an excess of serine in the growth medium reduced the productive replication of *C. trachomatis* serovar L2 (CT/L2) in HEp-2 cells by ∼2-logs, with a concomitant reduction in inclusion size. The mechanism by which excess serine reduces productive replication of CT/L2 was unknown but did not result from competition for the chlamydial branched-chain amino acid transporter (10). The parallels in inclusion phenotype and IFU reduction observed by us and Al-Younes *et al.* (9) led us to hypothesize that supra-normal levels of serine in growth media impacted CT/L2 by serving as a substrate for a serine deaminase.

Other than tryptophan synthase, which is not expressed during tryptophan sufficiency (11, 41, 42, 43), there are no other chlamydial enzymes predicted to deaminate serine. With rare exceptions (44), ammonia diffuses efficiently across bacterial and mammalian membranes (45, 46); therefore, we hypothesized that serine deamination by a host-cell encoded enzyme would also negatively impact chlamydial inclusion formation and IFU recovery. Our findings indicate that the host enzyme that deaminates l-serine to restrict the replication of *C. trachomatis* is serine racemase (SRR). To directly test the role for SRR in determining the effect of serine supplementation on *Chlamydia*, we ablated the SRR gene in HeLa cells using CRISPR/Cas9. Our results: (1) validate the hypothesis that host metabolism of supra-normal levels of serine limits the productive replication of CT/L2; (2) reveal SRR to be the major host enzyme that mediates the effect of supra-normal serine levels on CT/L2; and (3) identify the ammonia product of serine deamination as being most relevant for growth inhibitory effects on CT/L2.

Both human and murine SRR catalyze multiple reactions. Both enzymes catalyze the racemization of l-serine to d-serine and can deaminate l-serine efficiently. However, there are differences in their activities. Under optimal conditions obtained using purified enzyme *in vitro*, human SRR has a K_m_ for serine of ∼35 mM for the racemization reaction, and a k_cat_ of 35 (min^−1^). For the deamination reaction, the K_m_ for serine is 12 mM, with a k_cat_ of 183 (min^−1^) (20). These findings indicate that the deamination reaction is substantially favored over the racemization reaction (*ibid*). As described by Strisovsky *et al.*, murine SRR has a similar K_m_ for serine for both reactions, specifically, 3.8 mM serine for racemization *versus* 4.0 mM serine for deamination. However, for murine SRR the k_cat_ for deamination, 81 (min^−1,^), is twice that of racemization, 45 (min^−1^) (47). Comparing k_cat_/K_m_ values indicates that deamination is favored over racemization for both enzymes. It should be noted that these data were obtained using purified enzymes *in vitro*, leaving open the possibility that tissue-specific levels of co-factors may alter enzyme properties as described by de Miranda *et al.* (14). SRR’s function as a serine deaminase was established very early in its characterization, with the ascribed biological function of regulating d-serine levels by deaminating excess serine (14, 15). Given the importance of the glutamate/glutamine cycle in maintaining neuronal glutamate levels (48), we note that serine deamination also provides ammonia as a substrate for glutamine synthetase in the brain, and pyruvate for entry into the tricarboxylic acid cycle.

Our observations that serine deamination by human SRR restricts the replication of *C. trachomatis* likely has implications for previous findings by He *et al.* evaluating the role of serine in murine lung infections by the intracellular bacterium *Pasteurella multocida* (49). Like human SRR, murine SRR is expressed in epithelial tissue (32). He *et al.* demonstrated that infection of pulmonary epithelia by *P. multocida* rapidly decreased serine levels within murine lung tissue. Akin to our observation with *C. trachomatis*, when these authors exogenously supplemented serine levels *via* an intramuscular injection, they observed a significant decrease in *P. multocida* infectious burden, concomitant with decreased levels of pulmonary inflammation and increased survival of infected animals (*ibid*). While these authors did not evaluate the molecular mechanism by which serine restricted replication of *P. multocida*, biochemical data from Strisovsky *et al.* reveal the capacity of murine SRR to deaminate serine at least as efficiently as human SRR (47). When viewed in light of the observation that murine SRR is also expressed in epithelial tissue (32), our results raise the possibility that serine deamination by murine SRR also underlies their findings.

SRR’s capacity to produce ammonia by deaminating serine has an anti-microbial effect that can be exploited to protect against infections at mucosal surfaces. In this context, SRR’s expression in the gut is of interest because high dietary serine has been shown to correct gut microbiome dysbiosis in animal models (50, 51). It is possible that human serine-metabolizing enzymes, such as SRR, metabolize serine into products that restore microbiome homeostasis by differentially affecting bacterial genera, because of genus-specific differences in the capacity to assimilate ammonia and other products of serine catabolism (37, 52, 53).

The identification of ammonia as a host metabolite that curbs the productive replication of *Chlamydia* is likely relevant to previous observations indicating that high levels of glycine negatively impact CT/L2 IFU recovery (9, 10). While currently there is no mechanism ascribed to this observation, excess exogenous glycine can be metabolized by the glycine cleavage system (GCS) to generate 5,10-methylenetetrahydrofolate, carbon dioxide, and ammonia (54). Like SRR, the GCS is active in HeLa cells (55), rendering it a candidate to mediate the effect of excess glycine on CT/L2. A precedent for this hypothesis lies in the observation that *Streptomyces* use the GCS to generate ammonia as a diffusible antibacterial agent that can act at a distance from where it is produced (52).

While the antimicrobial properties of ammonia have been used in food storage for over 125 years (56), our results and those of Avalos *et al.* (52), bring to light the capacity of biologically-produced ammonia to restrict the growth of multiple bacterial species, including pathogens such as *Chlamydia*. The ammonia produced by human cells, or *Streptomyces* species, is a diffusible antibiotic that, in metabolic terms, is inexpensive to produce. However, while ammonia is simple to produce using glycine or serine as substrates, detoxifying it *via* assimilation into larger macromolecules can be relatively metabolically expensive. The enzymes used most frequently to assimilate ammonia are glutamate synthase (GOGAT), glutamate dehydrogenase (GDH), and glutamine synthetase (GS) (37, 53). Every molecule of ammonia assimilated by GOGAT and GDH requires NAD(P)+ to be reduced to NAD(P)H, while the assimilation of a single ammonia molecule by GS consumes a molecule of ATP (37). This increased metabolic demand likely impacts other bacterial processes needed for survival and proliferation. Thus, even bacteria, such as *Escherichia coli*, with multiple ammonia-assimilating enzymatic pathways, succumb to high levels of ammonia (52). Ammonia may be particularly deleterious to *Chlamydia* on account of common ammonia-assimilating enzymes being absent in *C. trachomatis.* A search of the annotated genome for CT/L2 along with a KEGG analysis suggests that the only chlamydial enzyme that can assimilate molecular ammonia is cytidine triphosphate synthetase (CTPS) (5, 57). While CTPS normally generates cytidine triphosphate (CTP) from uridine triphosphate using ammonia generated by a transglutaminase reaction, it can also use free (exogenous) ammonia (58). We note that *Chlamydia* cannot make the other three nucleotides (4), and can efficiently use host-provided cytidine (4, 59), rendering it somewhat puzzling that it has retained an expensive ATP-consuming mechanism to make CTP. It has been suggested that the evolutionary pressure to maintain CTPS may arise from its transglutaminase activity that provides the bacterium a source of glutamate (60). However, the chlamydial genome encodes for two symporters that can transport glutamate, and growth in glutamate increases the expression of both (61). We propose a second possibility arises from CTPS’ capacity to assimilate ammonia, which provides the bacterium a defense against ammonia-generating metabolic processes active in the host cell. Our hypothesis is consistent with the observation that CTPS is expressed throughout the chlamydial life cycle (57). The slow catalytic rate of CTPS (*ibid*), combined with the consumption of ATP for ammonia assimilation, likely permits this mechanism to be overwhelmed by a sudden increase in ammonia level imposed by the activity of host or bacterial serine deaminating enzymes. Transcriptomic and metabolomic studies will be of considerable utility in determining the molecular mechanism by which host serine deamination restricts the growth of *C. trachomatis*.

Regardless of the identity of the chlamydial enzyme(s) that assimilate ammonia, it is a reasonable conjecture that an increase in ammonia levels will require an increase in enzyme levels, and ergo, an increase bacterial susceptibility to antibiotics that impede protein synthesis. Consistent with this expectation, we observed that a modest increase in exogenous serine levels, which minimally impacted IFU recovery by itself, synergized with a sub-inhibitory concentration of doxycycline to reduce IFU recovery by several logs. Our findings join a growing body of work (62, 63, 64) indicating increased antibiotic sensitivity in response to alterations in bacterial metabolism with two important distinctions. In our approach, host cells catabolize the primary metabolite, serine, into a secondary metabolite, ammonia, that synergizes with an antibiotic. First, on account of serine deamination occurring in the host cell, it is unlikely to be prevented by a bacterial genetic change. Second, while there are ammonia/ammonium transporters, ammonia’s small size and lipophilic nature permit it to traverse membranes (45), rendering it difficult to completely block its entry. Interestingly, animal studies indicate high doses of serine are tolerated for extended times (65, 66), making it possible to test whether metabolic stress imposed by host serine deamination will synergize with an antibiotic to broadly overcome antibiotic resistance or tolerance in other intracellular pathogenic bacteria, particularly those that have rapidly gained antibiotic resistance or tolerance. Specifically for genital infections with *Chlamydia*, our studies raise the possibility of providing serine along with doxycycline directly at the site of infection with two beneficial effects: (1) A “topical” application will avoid perturbing other mucosal microbiomes, such as the gut microbiome; and (2) Decreasing the concentration of doxycycline needed for effective clearance will likely reduce bystander effects on healthy members of the genital microbiome.

## Experimental procedures

### Cell culture and media additives

HeLa (ATCC CCL-2) cells were cultured in DMEM (Sigma Cat# D6429, Millipore Sigma) supplemented with 10% calf serum (Gemini Bio Cat# 100-506, Gemini Bio). All reagents were of the highest grade available and purchased from Sigma (Millipore Sigma).

### *C. trachomatis* strains, and infections

The *C. trachomatis* strains L2/434/Bu (serovar L2; ATCC VR-902B) was used in this study. Infectious stocks were produced by passage in HeLa cells and purified using Optiprep gradients as described previously (6, 11, 67, 68). Infections were performed by spin-infection in SPG (10 mM sodium phosphate pH 7.2, 0.25 M sucrose, 5 mM L-glutamate) at the indicated multiplicity of infection (m.o.i) (typically 0.3) as described previously (*ibid*). Infectious unit (IFU) recovery was determined at 42 h post-infection (h.p.i) as described previously (*ibid*).

### Generation of HeLa ΔSRR cells

HeLa cells were co-transfected with a CRISPR/Cas9 plasmid specific for SRR (Santa Cruz Biotechnology Cat #sc-4022360-KO-2, Santa Cruz Biotechnology) and a corresponding homology-directed repair plasmid (Santa Cruz Biotechnology Cat #sc-4022360-HDR-2, Santa Cruz Biotechnology). Transfected cells were plated at a low density and subjected to puromycin selection at 0.5 μg/ml. Colonies obtained after 3 weeks of selection were individually picked, expanded, and screened for SRR expression by immunoblot using a rabbit monoclonal antibody specific to SRR (Abcam Cat# 200833, Abcam). Immunoblots were performed as described previously (67, 68). Two clones displaying a lack of SRR expression were pooled and used as HeLa ΔSRR cells, which were expanded without puromycin selection.

### HPLC analysis of l-serine levels

Cells were grown for 24 h in normal DMEM, or DMEM supplemented with serine. 2 × 10^6^ were pelleted, washed three times with PBS, and then immediately lysed using 200 μl of 1.5 M HClO_4_ and then neutralized with 100 μl of 2M K_2_CO_3_. 50 μl of each sample was used for HPLC analysis as described previously by Inoue *et al.* (32).

### Immunofluorescence and imaging

Control and infected cells were washed thrice with PBS, permeabilized and fixed, following which they were stained using a FITC-conjugated anti-chlamydial LPS antibody (Merifluor Cat # 500111, Meridian Bioscience, Inc) as described previously (32). Stained cells were imaged using Zeiss AxioObserver AX10 microscope equipped for inverted epifluorescence as described previously (32). Images were analyzed using Fiji v2. For infectious recovery, infections were performed in 24 well plates. 16 fields per well were imaged using the Agilent Biotek Cytation 1 Imaging Reader.

### D-serine quantification

Supernatants from HeLa and HeLa ΔSRR cells were filtered through a 0.2 μM filter, following which D-serine was measured using a D-Serine Colorimetric Assay Kit (Cosmo Bio Cat# CSR-CT-DSC-K01, Cosmo Bio USA), as directed by the manufacturer.

### Ammonia/ammonium quantification

Supernatants from HeLa and HeLa ΔSRR cells were recovered at 48 h post-plating, filtered through a 0.2 μM filter, following which ammonia/ammonium was measured using a colorimetric Ammonia Assay Kit (Sigma Cat # AA0100-1KT, Sigma), as directed by the manufacturer.

### Statistical analysis

Experiments were repeated a minimum of three independent times. Statistical significance was calculated with the Wilcoxon Rank Sum Test or Wilcoxon Signed Rank Test using MSTAT version 7 (69). Where indicated, means and standard deviations were calculated using Microsoft Excel.

## Data availability

The data that support the findings of this study are available from the corresponding authors, upon reasonable request by email to aaiyar@lsuhsc.edu and/or pdehon@lsuhsc.edu.

## Supporting information

This article contains supporting information.

## Conflict of interest

The authors declare that they have no known competing financial interests or personal relationships that could have appeared to influence the work reported in this paper.
