# Supplementary material for: Serine deamination by human serine racemase synergizes with antibiotics to curtail the replication of Chlamydia trachomatis
Source: J Biol Chem. 2024 May 6;300(6):107350. doi: 10.1016/j.jbc.2024.107350 (PMC11140210; doi:10.1016/j.jbc.2024.107350)
Supplement: Supporting Information [file mmc1.docx]

**Supporting Information Fig S1**: Pyruvate supplementation modestly impacts C. trachomatis inclusion size and IFU recovery in HeLa relative to HeLa ΔSRR cells. (A) HeLa and HeLa ΔSRR cells were infected with CT/L2 at an m.o.i of 0.3. Infected cells were exposed to DMEM containing 3.3mM pyruvate for 42 hours at which point primary inclusions were visualized as described in Fig. 3. At this concentration, pyruvate did not affect inclusion morphology in HeLa ΔSRR cells, while inclusions in HeLa cells were smaller. The scale bar at the bottom right corner of each image indicates a distance of 20 μm. (B) Primary infections of HeLa and HeLa ΔSRR cells, exposed to DMEM containing 3.3mM pyruvate, were assessed for recovery of infectious units (IFUs) at 42 h.p.i as described in the methods. Corroborating the smaller inclusion size observed in (A), IFU recovery from HeLa cells exposed to 3.3mM pyruvate was lower than IFU recovery from HeLa ΔSRR cells, albeit not significantly.
