# Supplementary figures and images for: Serine deamination by human serine racemase synergizes with antibiotics to curtail the replication of Chlamydia trachomatis
Source: J Biol Chem. 2024 May 6;300(6):107350. doi: 10.1016/j.jbc.2024.107350 (PMC11140210; doi:10.1016/j.jbc.2024.107350)

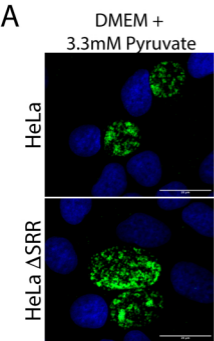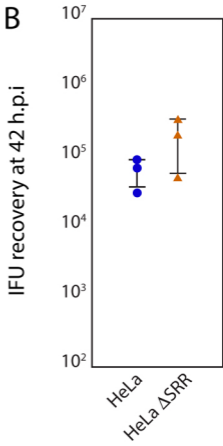

Supplement: Supporting Figure S1 [file mmc2.pdf]
