# Supplementary material for: Serine deamination by human serine racemase synergizes with antibiotics to curtail the replication of Chlamydia trachomatis
Source: J Biol Chem. 2024 May 6;300(6):107350. doi: 10.1016/j.jbc.2024.107350 (PMC11140210; doi:10.1016/j.jbc.2024.107350)
Supplement: Supporting Table [file mmc3.docx]

Table S1: Supplementation with L-serine dramatically reduces CT/L2 inclusion size in HeLa cells, but not HeLa ΔSRR

| **[L-serine] mM** | **HeLa** | **HeLa ΔSRR** |
| --- | --- | --- |
| 0.4 | 161.08 ± 34.3 μm^2^ | 314.78 ± 57.1 μm^2^  (p < 0.01) |
| 1.4 | 89.4 ± 41.8 μm^2^ | 284.13 ± 71.4 μm^2^  (p < 0.01) |
| 3.7 | 33.12 ± 13.3 μm^2^ | 227.07 ± 42.8 μm^2^  (p << 0.01) |

Inclusion area was measured from >50 primary inclusions fixed and stained at 42 h.p.i Images were obtained as described in the Materials & Methods, and area was estimated using Fiji v2. Where indicated, *p-values* were calculated using the Wilcoxon rank sum test, while comparing inclusions formed in HeLa vs inclusions formed in HeLa ΔSRR.
